# Material Extrusion Additive Manufacturing of Ceramics: A Review on Filament-Based Process

**DOI:** 10.3390/ma17112779

**Published:** 2024-06-06

**Authors:** Roberto Spina, Luigi Morfini

**Affiliations:** 1 Dipartimento di Meccanica, Matematica e Management (DMMM), Politecnico di Bari, 70125 Bari, Italy; 2 Istituto Nazionale di Fisica Nucleare (INFN)—Sezione di Bari, 70125 Bari, Italy; 3 Consiglio Nazionale delle Ricerche—Istituto di Fotonica e Nanotecnologie (CNR-IFN), 70125 Bari, Italy

**Keywords:** material extrusion, ceramic, additive manufacturing, mechanical properties

## Abstract

Additive manufacturing is very important due to its potential to build components and products using high-performance materials. The filament-based 3D printing of ceramics is investigated, revealing significant developments and advancements in ceramic material extrusion technology in recent years. Researchers employ several typologies of ceramics and binders to achieve fully dense products. The design of the filament and the necessary technological adaptations for 3D printing are fully investigated. From a material perspective, this paper reviews and analyzes the recent developments in additive manufacturing of material-extruded ceramics products, pointing out the performance and properties achieved with different material-binder combinations. The main gaps to be filled and recommendations for future developments in this field are reported.

## 1. Introduction

Additive manufacturing (AM) has emerged as a groundbreaking technology with several applications in numerous industries [1]. Traditionally, AM was predominantly associated with polymers and metals [2,3,4]. Recent advancements have extended its capabilities into ceramic materials, ushering in a new era of possibilities. Due to their excellent properties, ceramics are helpful in various sectors, such as the chemical industry, manufacturing, electronics, aircraft, and biomedical engineering. The material versatility is due to their outstanding properties, which include great mechanical strength and hardness, strong thermal and chemical stability, and the potential for impressive thermal, optical, electrical, and magnetic performance [5].

The main concept of material extrusion (MEX) additive manufacturing (ISO/ASTM 52900) requires continual feeding and heating of material within a moving nozzle at a temperature just above its melting point [6]. This process enables simple extrusion via the nozzle, molding layers along a preset path. After finishing a layer, the build platform or the extrusion head moves up or down, allowing for the deposition and adhesion of another material layer to the previous one. Support structures are incorporated into the procedure when complicated geometric features should be realized [7].

**Figure 1:**
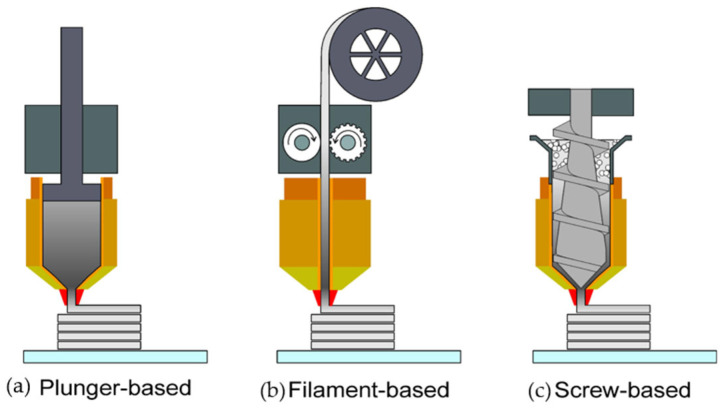
MEX processes are classified according to the extrusion method used: (**a**) filament-based, (**b**) plunger-based, and (**c**) screw-based extrusion [14].

**Figure 2:**
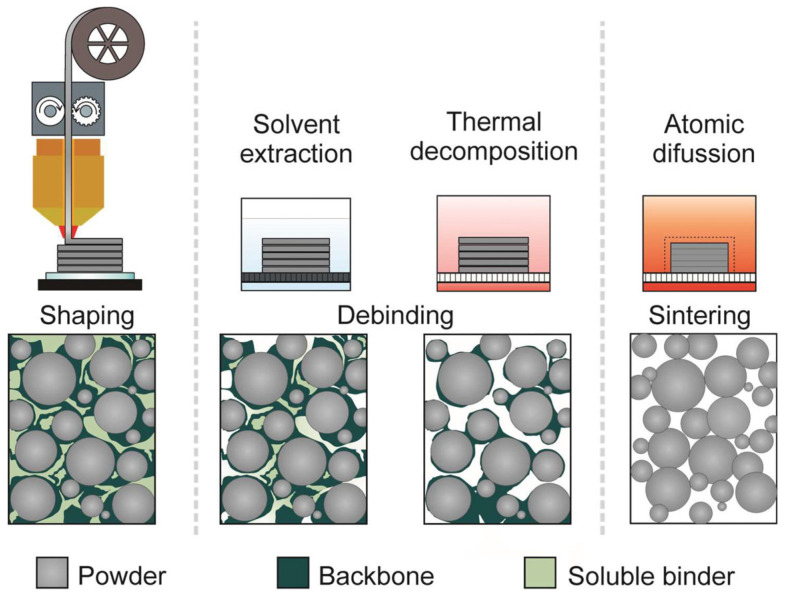
Shaping, Debinding, and Sintering process [14].

Based on the extruder used, MEX can be classified into three types (Figure 1): filament-based, plunger-based, and screw-based (Figure 1). When the feedstock material consists of sinterable powder, two more phases are incorporated into the production chain: debinding and sintering, with printing as the first stage, as shown in Figure 2 [8,9,10,11].

Fused Deposition of Ceramics (FDC), first introduced in 1995 by Danforth, is based on the patented Fused Deposition Modelling (FDM) technique developed by Stratasys Inc. (Eden Prairie, MN, USA) The FDC process was designed to fabricate ceramic components with filament produced with the same or similar feedstock used in injection molding [11]. Ceramics, as a class of materials, need help using traditional shaping processes due to their inability to deform plastically, limiting the provision of ceramics with complex shapes. The AM development revolutionizes this field by producing more precise, intricate ceramic components [12]. After printing, the result is referred to as a “*green part*”, which consists of sinterable powder (metals or ceramics), polymeric binders (main binder and backbone binder), and additives [13]. The ceramic powder concentration is typically up to 60% by volume. The binder system used to prepare feedstock is generally a mixture of one or more thermoplastic polymers. It is critical to carefully select the binder components and the production method, as these aspects might substantially impact the final sintered parts, even if they are eliminated during the debinding process [14].

The binder components can be divided into three categories [15]:

- *Primary binder*: This polymer, usually a thermoplastic, is the predominant constituent and serves as its primary element. In the debinding stage, it is the initial component that undergoes removal. Typically, a substance with a low molecular weight is selected to maintain lower viscosity, thereby preventing interactions with the powder and heavier components. Polyoxymethylene (POM), polypropylene (PP), polyethylene-glycol (PEG), low-density polyethylene (LDPE), thermoplastic elastomer (TPE), steric acid (SA), wax paraffin (PW), and polyolefin-based binders are examples of commonly used binders [9,16,17,18,19].
- *Backbone* (up to 50%): The second component remains unchanged throughout the debinding process. It contributes to the structural integrity of the portion after debinding. Thermal degradation of the backbone occurs either before or during the sintering process.
- *Additives* (up to 10%): To facilitate the even dispersion of powder particles within the binder, various additives are employed, including dispersing agents, compatibilizers, plasticizers, waxes, and stabilizers. These additions help prevent the segregation and clumping of constituents.

After debinding, the component is called a “*brown part*”. The primary binder is eliminated, resulting in a weight reduction of 4% to 10%, depending on the debinding process [16]. Solvent, thermal, and catalytic debinding are some fundamental ways. Solvent debinding is based on dissolving and diffusion phenomena, with treatment temperature and time depending on particle distribution and the size and form of the printed portion. Thermal debinding involves exposing the part to heat, which causes slight shrinking. Thermo-gravimetric analysis (TGA) can specify the temperature for binder removal and the corresponding weight loss%. In other cases, solvent and thermal debinding are performed together. Catalytic debinding is frequently used for POM-based filaments, taking advantage of POM’s vulnerability to degradation when exposed to nitric acid (HNO_3_).

During sintering, the residual polymer (backbone component) is removed. Solvent and catalytic are chemical-based, which has benefits and drawbacks to the specific debinding procedures. Catalytic debinding is harmful, whereas solvent-based debinding has a more negligible environmental impact. Thermal debinding consumes a lot of energy. Thermal and solvent debinding, on the other hand, can be modified to be more environmentally friendly. The brown component is sintered after removing the initial binder in a furnace. The residual polymer combusts, and the powder particles fuse during sintering to generate a wholly formed ceramic part known as a “*sintered part*”. The sintering temperature is approximately 70–90% of the melting point of the sinterable powder. Table 1 and Table 2 report an overview of ceramics obtained by filament-based MEX technology in terms of process parameters and mechanical properties.

The oversize produced during printing is an essential concern in manufacturing ceramic items utilizing this multi-step process. Removing the polymeric portion during debinding and sintering creates shrinkage in the final part’s volume and weight. As a result, accurate oversizing is required to attain the specified dimensions. This oversizing can be incorporated during the part design process or the slicing process. In the current research, a crucial emphasis is placed on optimizing printing parameters to achieve ceramic artifacts with a relative density of at least 95%. This objective is accompanied by the significant challenge of averting printing defects that could jeopardize the quality of the artifact. The aim is to attain high mechanical performance directly comparable to ceramics obtained through traditional manufacturing processes [17,18].

## 2. Material Extrusion of Ceramics

Numerous researchers review the AM of ceramic materials, analyzing several aspects and processes. Dadkhah et al. [39] focused on the challenges and advancements in the AM of ceramics compared to polymers and metals, giving a comprehensive overview of recent AM technologies for producing ceramic components with complex shapes. Bai et al. [40] studied advanced ceramics and the related AM fabrication processes, pointing out advantages, limitations, opportunities, and issues arising with each AM route provided. Datta and Balla [41] evidenced that the growth in the ceramic industry was rather slow due to several technical challenges, but it was necessary to produce fully dense defect-free ceramic components with complex geometry with AM technology. Their review classified the AM processes into powder bed-based, extrusion-based, photo-polymerization-based, and solid-based processes. Gonzalez-Gutierrez et al. [14] focused on AM of highly filled polymers with a particular emphasis on its application for the fabrication of metallic and ceramic components. A multi-step/indirect process, employing a sacrificial polymeric binder material to shape metallic and ceramic powder particles, removed the polymeric binder in subsequent treatments, bonding together the powder particles using a conventional sintering step. Walton et al. [42] described the AM methods with a shear field to the ceramic slurry or paste during forming. Using these methods, dense ceramic microstructures required the alignment of anisotropic particles via shear to tailor the ceramic microstructure independent of whether the AM ceramic was a bulk shape or a scaffold consisting of dense spanning rods. Romanczuk-Ruszuk et al. [43] reported the direct extrusion method, which gave the largest range of sizes of printed objects and enabled the scaling of processes in large-scale applications. The analysis focused on presenting ceramic materials using this method, especially for ceramic pastes. Rane and Strano [44] specified the main aspects of the feedstock AM processes for producing complex-shaped parts. The features and advantages of the AM processes were discussed with respect to materials and process steps.

**Figure 3:**
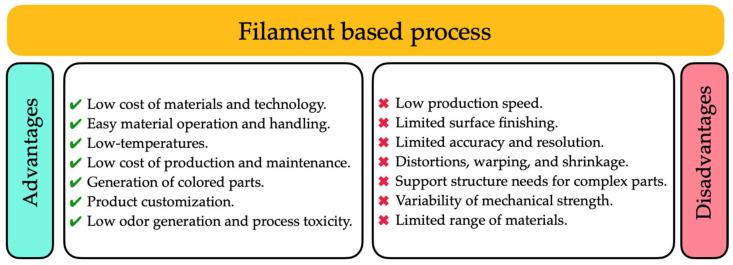
Advantages and disadvantages of filament-based MEX process [39].

Ceramics posed unique challenges in AM due to their high melting temperatures and brittleness. Various AM techniques developed in recent years were discussed, given the challenges of producing complex ceramic parts. The authors examined the AM method as MEX, which involved extruding ceramic materials in paste, filament, or pre-ceramic polymer filament forms through a nozzle to build a 3D structure. The success of MEX techniques relied on precise control of the extruded material’s rheological properties and solid loading to avoid porosity and cracks. The article also explored Fused Filament Fabrication (FFF), a widely used 3D printing method. FFF handles fused and non-fused materials, offering versatility in material choice. However, the quality of printed ceramic parts depended on various process parameters, such as layer thickness, building orientation, and filament width. Post-processing could be required to achieve a smooth surface. Despite these challenges, FFF was applied in several fields, including sensors, drug delivery, aerospace, and orthotics. The article touched on the economic aspects of AM, noting that the cost varied depending on the specific AM method and the desired product characteristics. While AM offered benefits in terms of customization and lower greenhouse gas emissions compared to conventional manufacturing methods, it was primarily used for prototypes or small-batch production. The article provided valuable insights into the progress and challenges of AM in ceramics, highlighting the potential for producing complex ceramic components with the proper techniques and optimizations. At the same time, disadvantages were also investigated. Figure 3 reports a schematic view of both. 

### 2.1. Al_2_O_3_

Nötzel et al. [21] focused on the realization of dense ceramic parts through FFF using a process chain analogous to injection molding. The key steps involved in this process were as follows:

1. *Material Selection*: The study starts with selecting materials, including fillers, binders, and surfactants.
2. *Compounding and Rheological Characterization*: After material selection, compounding and rheological characterization are carried out to ensure the materials’ proper mixture and flow properties.
3. *Filament Extrusion*: The next step is filament extrusion, where the material mixture is formed into filaments suitable for 3D printing.
4. *Feedstock Printing*: These filaments are then used as feedstock for 3D printing, where they are deposited layer by layer to create the desired ceramic structures.
5. *Thermal Post-Processing* (Debinding and Sintering): After printing, thermal post-processing involves debinding to remove the binders and sintering to achieve the desired density.

The selected ceramic material was submicron-sized alumina (Al_2_O_3_), known for its high sintering activity and the ability to achieve densities exceeding 99% at moderate sintering temperatures (1400 °C). The alumina’s specific surface area and particle size were measured using the Brunauer–Emmett–Teller (BET) method. A partially water-soluble polyvinyl butyral (PVB) binder was utilized as the backbone polymer, and polyethylene glycol (PEG 4000) was used as a low molecular weight plasticizer. Stearic acid was employed as a surfactant to facilitate the coupling between the ceramic and polymer components. The results indicated that feedstock with 55 vol.% alumina was too brittle for extruding and printing, while a reduced 50 vol.% alumina load could be successfully handled. The printed green body structures did not exhibit delamination or warpage. The printing parameters for this new PEG/PVB-based feedstock system were found to be like those for the established wax/PE system from [19,44]. After printing, structural design quality and accuracy were evaluated with a complex clamping test structure with asymmetric serrated profiles. The surface quality and dimensional accuracy were assessed. The study concluded that, with slight adaptations to the feedstock recipe, dense and warpage-free alumina parts, including complex structures, could be realized through FFF printing and subsequent thermal post-processing. The sintered alumina parts achieved a density of approximately 98% of the theoretical value. Exploring alternative surfactants and lower molecular weight PEG could enhance plasticization further and reduce sinter shrinkage.

**Figure 4:**
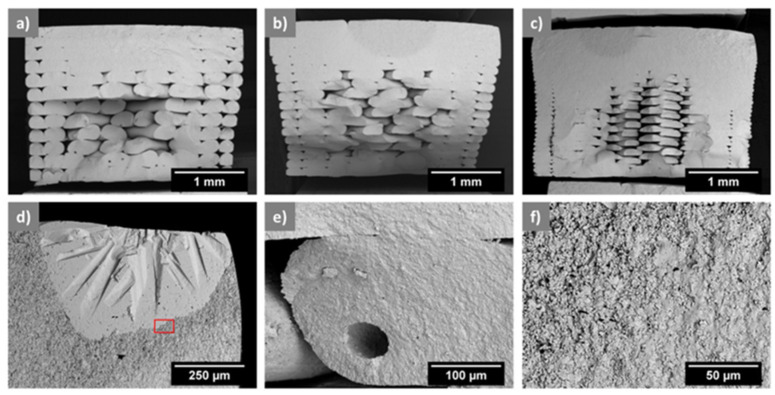
SEM study of the fracture surface of sintered bars with layer thicknesses of (**a**) 0.3 mm, (**b**) 0.2 mm, (**c**) 0.1 mm, (**d**) anomalous alumina grain, (**e**) pore inside the filament, and (**f**) boundary between intergranular and transgranular fracture behavior (BSE) [22].

Orlovská et al. [22] investigated the flexural strength of alumina through three-point bending tests. A thermoplastic composite filament with a solid content of 50% and an average diameter of 1.75 mm was created with high-purity alumina powder (99.99%) from Sumitomo Chemical Co., Ltd., Tokyo, Japan. The polymer matrix to prepare the filament was chosen from well-known binder systems used in ceramic injection molding. This desired composition allowed for chemical debinding using cyclohexane. Bars with nominal dimensions of 4 × 3 × 45 mm^3^ acceptable for standardized mechanical property testing were realized. The bars were created with three layer heights (0.1 mm, 0.2 mm, and 0.3 mm) and a rectilinear infill density of 65%. A8 DIY desktop 3D printer (Shenzhen Anet Technology Co., Ltd., Hangzhou, China) was used to realize the specimens after slicing them with the Slic3r application. The extrusion and bed temperatures were set at 240 °C and 60 °C, whereas the printing speed was between 10 and 15 mm/s. Two-stage consolidation was applied: solvent debinding in cyclohexane and heat debinding up to 850 °C, then sintering for one hour at 1600 °C. The bulk density of the bar specimens was determined, and the relative density was calculated based on the bulk densities and the theoretical density of alumina (3.99 g/cm^2^). The surface quality was evaluated using a laser scanning confocal microscope, and the elastic modulus was determined using an impulse excitation technique. Flexural strength was measured using a three-point bending configuration, and the macroscopic microstructure and fractographic observation were conducted using an optical microscope and scanning electron microscope (SEM). The relative density of the printed specimens ranged from 46% to 49% after debinding and increased to 86% to 89% after sintering. The elastic modulus correlated well with bulk density, with specimens having elastic moduli between 300 and 350 GPa. The flexural strength decreased from 300 to 200 MPa with an increase in the layer height from 0.1 to 0.3 mm. The microstructure analysis revealed the presence of defects such as abnormally large grains, internal pores, and abnormal grain growth (Figure 4). These findings suggested that the layer height used in MEX and the printing strategy significantly impact alumina specimens’ mechanical properties and microstructure. Adjusting the printing parameters and debinding steps could help improve the quality of printed alumina parts by obtaining green bodies without internal voids, resulting in sintered parts with high relative density.

Gorjan et al. [19] focused on the preparation of thermoplastic Al_2_O_3_ filaments, utilizing ethylene vinyl acetate (EVA) and stearic acid (SA) as an organic binder. The concentration of SA was found to substantially impact the rheological properties of the thermoplastic feedstocks and the printing behavior of the resulting thermoplastic filaments. Specifically, a significant change in rheological behavior occurred when the SA on the surface of the powder reached apparent saturation. Above a concentration of 3.5% of SA, a yield point became detectable, and the shear-thinning effect became more pronounced, as evidenced by parameters from the Herschel–Bulkley model. Such changes in flow behavior were so substantial that different rheological models were required to describe the flow behavior for feedstocks with lower and higher SA amounts. The Cross model yielded a better fit for feedstocks with lower SA content (<4.2%), while the Herschel–Bulkley model was necessary for feedstocks with higher SA content. A theoretical prediction of the force required to extrude the filaments through the printing head of a filament-based MEX printer was also proposed. The applicability of specific models described by Ramanath et al. [45] and Turner et al. [46] for determining the printability of ceramic filaments was discussed. These models became inaccurate when SA reached apparent saturation due to a slipping effect associated with higher SA content. Furthermore, the flexibility of ceramic filaments decreased as the SA content increased. Nevertheless, printability improved with very high SA contents, allowing for the extrusion of vase structures across a wide temperature range of the heated printing nozzle. A model was proposed to elucidate the influence of SA on the feedstock’s flow behavior and the filaments’ flexible properties. This model posited that SA initially forms a monolayer coating on the surface of ceramic powder. Once saturation was reached, excess SA started to create regions or networks within the EVA matrix, explaining the increased yield point in feedstocks with higher SA content. Similar behavior was reported previously in filaments based on tricalcium phosphate, EVA, and SA. The study concluded that the described binder system allowed for printing thin-walled alumina structures that could be successfully debinded and sintered. However, mechanical testing revealed relatively low mechanical properties of the sintered ceramic, with printing defects and pores at grain boundaries being identified as potential areas for improvement in mechanical values.

Orlovská et al. [23] further investigated MEX processes by expanding their revision research. A high-purity alumina powder composite filament and a polyolefin-based binder system were employed to realize several shapes, improve printing parameters, and examine bulk densities and shrinkage. A filament made by fusing alumina powder and a binder was used for the MEX process. Micro-computed tomography (CT) was employed to gain insights into the internal structure of the printed objects, yielding significant information on porosity and shrinkage phenomena. The Archimedes technique evaluated the bulk density. The MEX process offered a distinct advantage over conventional ceramics manufacturing methods due to its precise control over internal structures, mitigating errors related to subsequent binder removal. Optimizing slicing root control during printing and debinding processes was highlighted, particularly for asymmetrically designed ceramic objects. Effective control over void generation during printing and ensuring strong inter-layer adhesion could enable filament-based MEX to produce deformation-free, highly dense solid ceramic objects.

**Figure 5:**
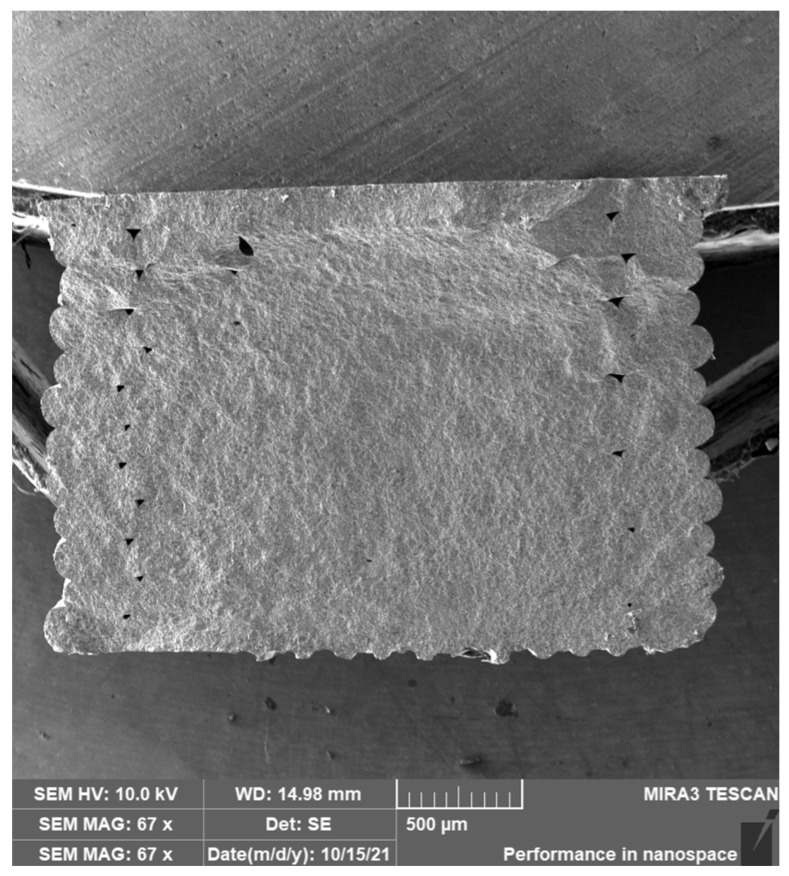
SEM study of the fracture surface of bending sample with internal infill 100% [26].

Truxová et al. [26] comprehensively investigated using MEX technology to produce alumina material. A thorough analysis was performed using TGA, Fourier transform infrared (FT-IR) spectrometry, energy-dispersive X-ray (EDX) analysis, and melt volume rate (MVR) measurements. The binder system comprised 48 vol.% while the alumina particles constituted 52 vol.%. The thermal decomposition process was initiated at 181 °C, with the peak of weight loss observed at 381 °C. To achieve desirable results, adjustments were made to the printing parameters. However, the chemical debinding introduced sensitivity to cracking and delamination, necessitating removing a substantial portion of the binder in an acetone bath. Subsequent thermal debinding and sintering processes were employed to complete the fabrication. After post-thermal debinding, the parts exhibited pronounced brittleness. The relative density achieved at the end of the process was 99.54% at 100% infill, with the highest porosity detected in the perimeter regions, as shown in Figure 5. The material’s hardness linearly correlated with infill percentage, culminating at 2428 ± 209 HV10 (23.81 GPa). A three-point bending flexural test revealed no discernible dependence on infill density, with flexural strength measured within the range of 316 MPa. Shrinkage emerged as a significant characteristic of the composite system, with the part’s weight decreasing by approximately 22.6 wt.% after the sintering process. These mechanical properties and relative densities were comparable with conventionally manufactured components.

**Figure 6:**
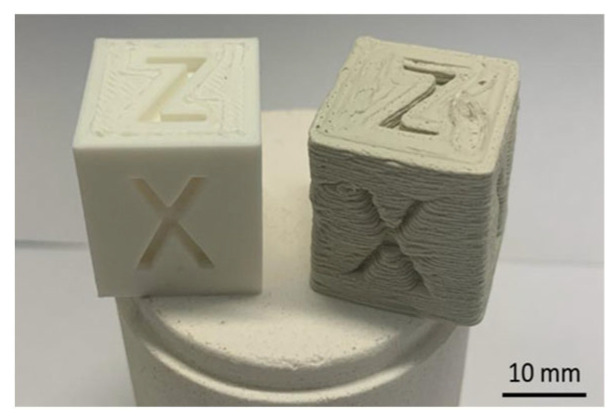
Print quality of calibration cubes in PLA (**left**) and 50% Al_2_O_3_/50% PLA (**right**) [24].

Smirnov et al. [24] investigated a solvent-free method for producing ceramic-polymer filaments for 3D printing using the FFF technique. The focus was on filaments with a polymer matrix based on polylactide (PLA) and a ceramic component ranging from 50 to 70 vol.%. The structural composition of the ceramic-polymer mixtures was initially examined through FT-IR spectroscopy. The results confirmed that the selected filament production method did not lead to significant structural changes or the introduction of impurities into the composition. Rheological studies of the ceramic-polymer compositions revealed that filaments containing up to 60 vol.% Al_2_O_3_ were suitable for extrusion-based 3D printing. However, only the 50% Al_2_O_3_/50% PLA filament could be successfully printed due to limitations with higher ceramic content filaments such as brittleness, irregular surface quality, and significantly increased viscosity (15 times that of PLA), which hindered the printing process. Additives were added to reduce viscosity, allowing the flexibility required for extrusion and improving accuracy in diameter. Three-dimensionally-printed objects produced from the 50% Al_2_O_3_/50% PLA filament exhibited lower print quality than commercially PLA filaments (Figure 6). Future research efforts should be addressed to optimize printing parameters, reduce defects in ceramic-polymer samples, and find techniques to remove the polymer binder and sinter the ceramic specimens to achieve high-density ceramic objects.

Tosto et al. [25] evaluated a tool-free AM technique to produce ceramic objects, specifically prototypes and small quantities. The study identified several key findings and limitations. The functional AM ceramics faced challenges in achieving high density. The sintered samples exhibited an alumina crystalline phase with an average density of 3.80 g/cm^3^. Despite significant research efforts, achieving dense alumina ceramics for widespread industrial use required further experimental work and substantial financial investment. The printing and sintering parameters notably influenced the thermo-mechanical properties. The sintered ceramic parts produced through this AM technique exhibited a tensile strength of 232.6 ± 12.3 MPa and a Vickers hardness of 21 ± 0.7 GPa. The thermal conductivity at room temperature averaged 21.52 ± 0.02 W/(m × K). The values obtained with AM were lower than those achieved through conventional processes due to voids and imperfect interlayer bonding. Filaments with tight dimensional tolerances were suggested to maximize density and mitigate shrinkage, manage the flow ratio, and assess the impact of ceramic powder content and moisture on the degradation of organic components. The long thermal cycles required for de-binding processes, owing to high levels of organics in AM parts, contribute to significant shrinkage during sintering. Highly filled ceramic filaments were the best solution for cost-effective FFF fabrication. 

**Figure 7:**
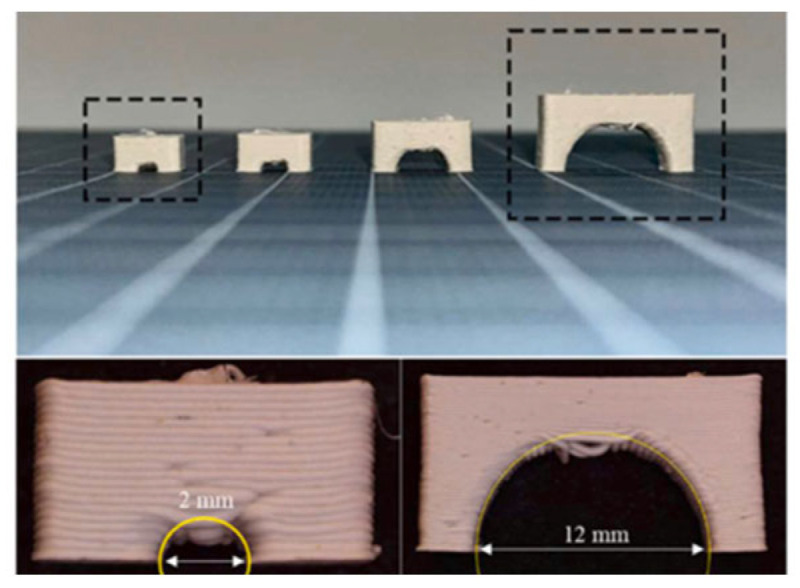
Test specimens to evaluate the printability of ceramic filament. The close-up view shows printing defects [20].

Hadian et al. [20] investigated crucial design/processing parameters such as the minimum build angle, printability of arches, minimum wall thickness, and shrinkage after debinding and sintering on a series of geometric test specimens printed using a filament-based extrusion head. The test specimens were the basis for establishing initial design rules specific to the MEX and post-processing of alumina parts. The aim was to demonstrate how the findings from simple geometric structures could be applied to design complex components made from alumina without additional support structures. Various aspects of ceramic MEX were investigated, covering the printing and post-processing stages. Test specimens were chosen to assess the manufacturability of different design features crucial for constructing a multi-flow nozzle and an alumina-based heat exchanger. Prior studies on MEX polymers influenced these design features. For overhang samples, the study explored a range of build angles and wall thicknesses, considering the effects of gravity on thin structures. Arch-shaped geometries were analyzed for their diameter variations, while thin-walled structures were studied for shape stability (Figure 7). Cuboids were used to measure shrinkage in different directions during debinding and sintering. One key finding was related to the minimum build angle for overhangs. After printing, samples with less than a 5° deviation from the digital model were considered successful. According to the study of the simple geometries, walls with thicknesses of 1.0 and 1.5 mm were employed for freestanding overhangs with building angles of 50° and 40°, respectively. Solvent debinding appeared to alleviate some issues with overhang structures, suggesting a potential strategy for addressing internal stresses generated during printing. Wick debinding led to notable deformations during sintering due to gravity-induced creep. Results revealed that samples shrank anisotropically during the debinding and sintering process, with a total shrinkage of 19.3 ± 0.2% in the plane (*x*-/*y*-axis) and 28.6 ± 0.4% in the printing direction (*z*-axis).

### 2.2. ZrO_2_

**Figure 8:**
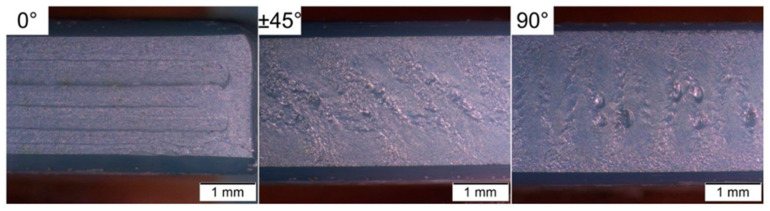
The bottom layer of sintered bending specimens with different raster orientations highlights principal defects found by [31].

Cano et al. [31] investigated flexural characteristics using TZ-3YS-E tetragonal zirconia powder stabilized with 3 mol.% yttria [47]. The average particle size of the powder was 90 nm, and the specific surface area was 7.2 m^2^/g. A multi-component binder system, composed of a commercial thermoplastic elastomer compound (TPE) and polyolefin grafted with a polar component, was used to improve adherence to the powder. The effects of raster orientation on the characteristics of MEX zirconia parts were investigated. Bending bars with raster orientations of 0°, ±45°, and 90° were manufactured, and the quality of the ceramic feedstock and sintered parts were evaluated. The powder’s incorporation into the organic binder changed the rheological characteristics and thermal degradation behavior. During the rheological measurements of the feedstocks, pressure oscillations were seen at a shear stress of roughly 0.25 MPa, resulting in pores inside the roadways during MEX shaping. MEX shaping produced three categories of defects: inter-road faults, under- and over-extrusion defects, and shearing-off defects of the deposited material in the first layer (Figure 8). The variation in filament diameter changed the shape and orientation of these flaws, which, in turn, affected the specimens’ bending strength. The bending behavior of the parts was predominantly controlled by the first layer’s quality, with interfaces normal to the applied stress, poor bed leveling, filament diameter changes, and shearing-off of deposited material resulting in strength value fluctuation. Parts produced with a 0° orientation were less reliant on these faults than those printed with a 90° orientation. When the tensile side was defect-free, pores within the specimen caused failure. The orientation of infill roads relative to the applied loads was considered to build robust and dense components using MEX. Flaws could be reduced by minimizing inter-road faults, employing filaments with strict dimensional tolerances, and regulating over-extrusion.

Nakai et al. [48] studied the properties and capabilities of alumina-toughened zirconia (ATZ) and AM zirconia ceramics, focusing on their use in dental applications. The comparison of these ceramics to conventionally subtractive manufactured (SM) zirconia ceramics was completed, considering that AM zirconia ceramics had a phase composition of 86/88 wt.% of tetragonal zirconia (t-ZrO_2_), closely resembling the SMZC composition. However, AM zirconia ceramics contained approximately 20 wt.% of Al_2_O_3_ in its phase composition. Biaxial flexural strength tests demonstrated that AM zirconia ceramics exhibited strength comparable to their SM counterparts. AM zirconia ceramics were characterized by superior biaxial flexural strength compared to SM 3Y-TZP (3 mol% yttria-stabilized tetragonal zirconia polycrystals). Compared to AM 3Y-TZPs and ATZ, microstructural examination of SM zirconia revealed more frequent porosity. Differences in alumina concentration between SM and AM zirconia were revealed by EDX spectroscopy. AM ATZ and zirconia ceramics had crystal structures and microstructures like the SM counterparts, with increased biaxial flexural strength.

Nötzel et al. [36] developed a comprehensive process chain for producing sintered zirconia components. This process involved several steps, including compounding FFF printing, debinding, and sintering. Selecting specific parameters to ensure the quality of the FFF printing was crucial. Previous research [21,49] indicated that the filament’s diameter should remain within a strict tolerance of ±0.1 mm to minimize the swelling of highly filled filaments. Optimized printing parameters were identified, but their process window was very narrow—deviations from the optimal values led to material extrusion problems, filament fracture, or undesirable thermal effects. The printed samples underwent thermal post-processing after printing, which involved debinding and sintering. Careful attention was paid to the temperature profiles to minimize internal stress and ensure proper sintering. A controlled heating and cooling rate characterized the sintering process. The resulting sintered zirconia parts exhibited various structural features, including boreholes and cantilever arms. These part densities were analyzed using the Archimedes method, realizing sintered parts with a density of approximately 99% with successful void filling. Microscopic examination of the sintered discs revealed the presence of triangular voids, which were not perfectly periodic due to the changing printing direction with each layer. These voids were partially filled due to variations in filament diameters and filament trace distances. The overall sintered structure exhibited fine grain size and good deagglomeration, indicating successful compounding and wetting. The surface quality of the green zirconia parts was examined, and well-packed filaments without visible defects were found. The study demonstrated the feasibility of using FFF printing to produce high-density zirconia parts, providing an alternative to traditional powder injection molding processes. The results indicated that FFF could offer design flexibility and rapid prototyping for zirconia parts, although some mechanical property trade-offs might be involved compared to injection molding. Future research directions include exploring higher ceramic load compositions and improving the printing of complex and fine structural features while reducing voids to enhance mechanical properties.

Hadian et al. [34] investigated the area of AM of massive zirconia structures. A material extrusion-based technique was investigated with filaments made of zirconia powder and an ethylene-vinyl acetate (EVA) binder. The primary objective was to streamline the entire production process for massive ceramic structures, from 3D printing to debinding and sintering. The inquiry started with making filaments using zirconia powder partially stabilized by yttria and an EVA-based glue. To customize the filament’s characteristics, various EVA grades were thoughtfully selected. A consumer filament-based MEX machine was employed. Extensive optimization was executed on critical variables such as nozzle temperature, printing speed, and extrusion multiplier. Dynamic infill techniques were also investigated to increase the flow rate for the initial layers, reduce errors, and enhance printing quality. A multi-stage debinding procedure encompassing solvent removal and thermal debinding was adopted, followed by sintering to provide the necessary structural integrity. Biaxial flexural strength tests were conducted on the sintered zirconia disks to evaluate their mechanical characteristics. These tests revealed impressive results, with a biaxial flexural strength of 90 MPa and a modulus of 5.7. The material exhibited a shrinkage value of approximately 23%. Optical and SEM microscopy allowed the fractographic study to examine the fracture surfaces and provided valuable insights into the material’s behavior under stress.

Hadian et al. [35] conducted a study involving Ceramic Injection Molding (CIM) commercial binder compositions to create feedstocks with 45 vol.% ceramic powder loading for MEX of zirconia samples. A screw-based printing head was employed to produce dense disk structures for subsequent mechanical ring-on-ring analysis to address the rigidity of ceramic strands. A comparison was made with disks printed using a commercial filament (Fabru GmbH, Switzerland) and cold isostatic pressed disks made from pellets made with 3 mol% yttria-stabilized zirconia powder with several binders. Among the selected commercial binders, feedstocks based on Embemould K83G and K84G exhibited phase separation during processing and were excluded from 3D printing experiments. Instead, Embemould CC and M-based pellets were utilized. The feed rate setting for screw-based printing heads differed significantly from filament-based counterparts. Extensive investigations of the printing parameters were conducted using the feedstock based on the Embemould CC binder composition, which was identified as the most performant. The slope at low flow rates was observed to exceed that at higher flow rates. Higher temperatures reduced material output due to increased leakage flow and partially melted feedstock pellets bridging near the hopper area. The study demonstrated that material output could be maintained consistently even with low-cost commercial pellet extruders by adjusting the multiplier before printing parts. During solvent debinding, the integrity of the printed disks based on the Embemould M binder composition was compromised due to the delamination of the printed layers. These samples were not further investigated. By employing a post-printing process involving solvent debinding, partial debinding, thermal debinding, and sintering, zirconia disks were successfully manufactured without introducing cracks and blisters. Experiments with different mass loss and shrinkage confirmed that the ceramic powder content in the Fabru filament was higher than feedstock fabricated with the Embemould CC binder composition. For mechanical ring-on-ring analysis, all pressed and printed disks exhibited a low Weibull modulus, a phenomenon well documented in the literature, primarily attributed to sample setup and surface roughness. However, disks produced using cold isostatic pressing (CIP) and 3D printing with Fabru filaments yielded similar biaxial strength values. The slightly lower sintered density in 3D-printed samples could explain the tendency towards lower strength values. Disks fabricated with Embemould CC binder composition, which experienced delamination during the debinding process, displayed significantly lower sintered density and biaxial strength. Fractography studies revealed that failures originated near the loading ring area, indicating uneven stress distribution during ring-on-ring measurements. This uneven stress distribution was considered the cause of the low Weibull modulus observed in all tested samples. SEM fractography on sintered samples confirmed poor fusion between the printed layers as the main reason for inferior mechanical performance, particularly in pieces fabricated with the Embemould CC binder composition. The study suggested commercial CIM binder compositions can be employed in MEX ceramic feedstock fabrication. However, lower mechanical strength compared to conventionally shaped ceramics should be anticipated. The proposed remedy is to tailor the CIM binder composition to achieve improved fusion during printing.

Guan et al. [33] investigated the effect of binder content and material weight on the mechanical properties of filaments. The first zirconia powder utilized in the investigation had a specific surface area of 7.2 m^2^/g and contained 3 mol.% yttria. A multi-component organic binder was developed using various formulations of HDPE, SEBS, paraffin wax, SA, and dibutyl phthalate. Filaments with a diameter of 1.75 ± 0.05 mm were created through mixing and extrusion. Commercial 3D printers were used for fabrication, focusing on mechanical quality and printability. The organic binder content considerably influenced tensile stress and elongation at break. Higher organic binder content increased flexibility and toughness, benefiting FFF continuous extrusion.

**Figure 9:**
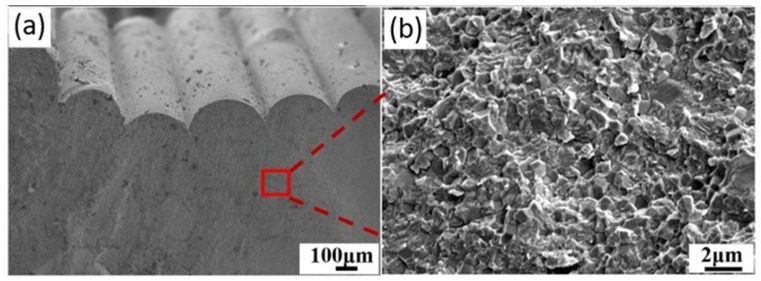
(**a**) SEM study of the fracture surface of the bending sample near the side of the bending sample. (**b**) enlarged image of the fracture surface [33].

A reduced powder loading occurred, potentially compromising the mechanical qualities of the ceramic component. The study researched effectiveness of 3D-printed complex structures such as wheels, gears, porous structures, and spheres, indicating the appropriateness of the filaments developed for using MEX to construct intricate ceramic components. Because of the relatively high amount of organic binder in the green bodies, a separate debinding operation was necessary before final sintering. A two-step debinding technique was introduced, involving the removal of paraffin wax using kerosene followed by heat debinding. This approach substantially minimized deformation and cracking while eliminating soluble binders. As a prevalent fault in the printed components, inadequate interlayer bonding resulted in pores or fractures and triangular voids at the junction of extrusion pathways. These defects substantially impact sintered materials’ density and flexural strength. These issues were alleviated by adjusting printing parameters and increasing nozzle temperature. Data on the density, shrinkage, Vickers hardness, and bending strength of rectangular bars printed with different solid loading percentages were collected. The highest solid loading of 82 wt.% resulted in a relative density of 99.1% and a bending strength of 492.8 ± 40 MPa (Figure 9), demonstrating the potential of FFF for zirconia ceramic production.

**Figure 10:**
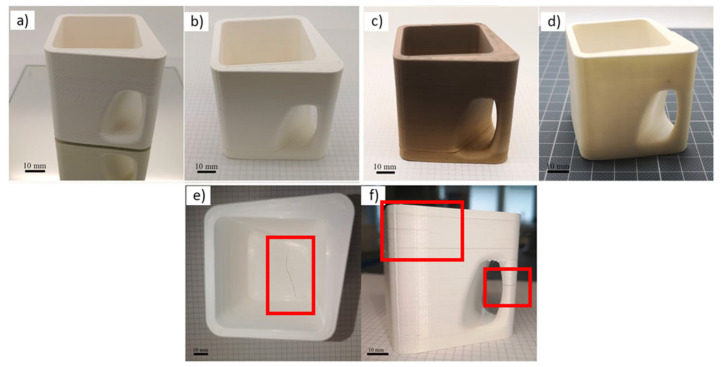
Picture of the printed 3D cup using YSZ Fabru filament after (**a**) shaping, (**b**) solvent debinding, (**c**) wick debinding, and (**d**) sintering. (**e**,**f**) show a cup printed using SiCeram filament showing cracks in the bottom area and between layers after solvent debinding [32].

Clemens et al. [32] studied the printing and post-processing behavior of commercial YSZ filaments from SiCeram GmbH, PT+A GmbH, and Fabru GmbH for the 3D printing of a cup. A cup had different wall thicknesses, which resulted in different solvent-debinding behaviors and deformation during thermal treatments. The density of the filaments was initially analyzed by a He-pycnometer (Ultrapyc 500, Anton Paar, Graz Austria), and the filament diameter was measured with a caliper. The flexibility of filaments was evaluated as the minimum bending radius to which a filament could be bent before it fractured. The fracture surface of the filaments was studied using SEM. To investigate the homogeneity of the YSZ filaments, a Rosand RH7 capillary rheometer (Netzsch GmbH, Hanau, Germany) was used by filling a mass of 140 g heated to 150 °C at a shear rate of 6000 1/s. A rotational rheometer was then used to analyze the flow behavior of the three thermoplastic YSZ materials, investigating the rheological behavior at three temperatures (120, 140, and 160 °C). The binder removal and sintering were analyzed on the commercial filaments after solvent debinding and after sintering. The solvent debinding was performed in an acetone bath at room temperature for 48 h for all filaments. An Ender 5 Pro (Creality Inc., Shenzhen, China) with an E3D Hemera direct kit was used to print the open-source cup design. The size of the printed samples was tuned to achieve the correct dimensions after sintering. All selected filaments were printed with a layer height of 0.2 mm and an extrusion width of 0.75 mm. The PT+A filament was not chosen for the printing phase because its characteristics were similar to those of SiCeram. The SiCeram filament posed challenges because only one sample was successfully produced after using 1 kg of raw material by cracking issues during the sintering process (Figure 10e,f). The viscosity of printing materials was crucial to investigate for successful 3D printing, as high viscosity led to buckling and filament abrasion. Viscosity data were valuable for selecting the right printing temperature for a new filament. Regardless of whether ceramic filaments were brittle or flexible, printing ceramic objects like cups was feasible. However, brittle filaments were characterized by a high rejection rate due to filament fractures, making it challenging to continue printing without introducing defects. Shrinkage in ceramic parts was consistent for outer dimensions, and knowing the shrinkage values of ceramic filaments was important. Shrinkage variation occurred in different directions due to ceramic powder characteristics, infill orientation, and printing technology. It was advisable to investigate filaments thoroughly before starting ceramic 3D printing, utilizing rheology to fine-tune printing parameters and understand shrinkage patterns for precise design.

**Figure 11:**
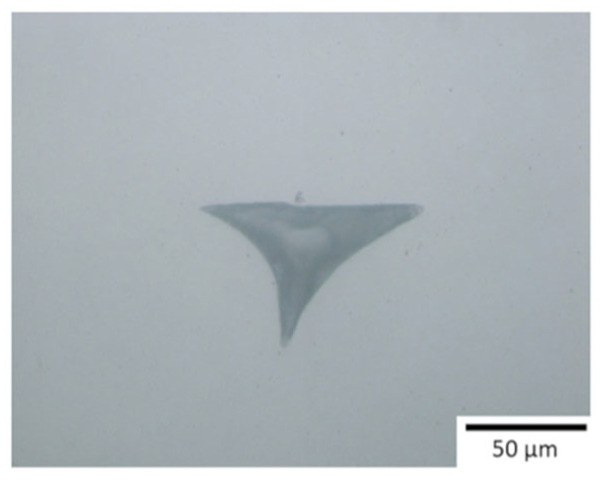
The printing process generated residual pores after MW 25 °C/min (adapted from [30]).

Petit et al. [30] examined the combination of MEX and Microwave (MW) sintering processes to build highly dense 3Y-TZP (3 mol% yttria-stabilized zirconia) ceramic samples with respect to conventional (CV) sintering processes. The 3Y-TZP filament used for sample preparation, supplied from Zetamix (Palaiseau, France), comprised a proprietary mixture of 50% 3Y-TZP powder and 50% organic binders. At the end of the process, the MW-sintered pellets achieved densification levels from 93.9% to 97.7%, with heating rates ranging between 25 and 80 °C/min, comparable to 96.8% produced by CV sintering (Figure 11). Dilatometric curves for MW sintering exhibited identical linear shrinkage behavior of CV sintering. Despite printing at 100% infill density, the printing process caused the formation of inner pores. Improving the printing path during the slicing allowed the manufacture of dense ceramic items. A MW thermal cycle’s power and temperature profiles showed a high correlation between the set and measured temperatures, demonstrating perfect control of the heating cycle. The study also linked 3Y-TZP dielectric properties to changes in MW power absorption, which enhances dielectric loss at 500 °C.

Bhandari et al. [29] presented an approach demonstrating the feasibility of ultra-rapid debinding and sintering [50,51] for complex 3YSZ components realized with MEX. This process resulted in fully dense components with tailored microstructures and nanometric grain sizes. The components were completely free of cracks, even at the microscopic level. A commercial YSZ white zirconia filament from Zetamix (Palaiseau, France) containing 50 vol.% YSZ powder embedded in an organic binder was used to achieve these results. A gyroidal pattern was employed to produce the intricate geometry of test components, which was impossible with conventional fabrication techniques. This pattern possessed high porosity and surface accessibility and a high strength-to-weight ratio, making it useful in various applications, including structural weight reduction, biomedical, and aerospace. The debinding and sintering process involved partially chemically debinding the green bodies in acetone, with about 50% of the organic binder being removed. Ultrafast high-temperature sintering (UHS) was applied to these partially debinded components. A specific setup with graphite felt clamped between two steel plates and connected to a DC power source was used for UHS. The sample was placed in the center of the felt, and different current levels and holding times were applied to induce Joule heating. Conventional sintering experiments were also conducted to compare air and argon atmospheres. The results showed that UHS was a highly efficient process, combining thermal debinding and sintering in a single step that takes only a few tens of seconds with a final output of relative density of 99% with a current of 34A and a duration of 120 s. The time of the chemical debinding step can also be reduced to 30 s, achieving a relative density of 98%. Finally, the final density and microstructure of the sintered components were controlled by adjusting the UHS current and time. Optimized UHS cycle yields comparable hardness value (15 GPa) to traditionally debinded and sintered materials.

### 2.3. Other Ceramic Materials

**Figure 12:**
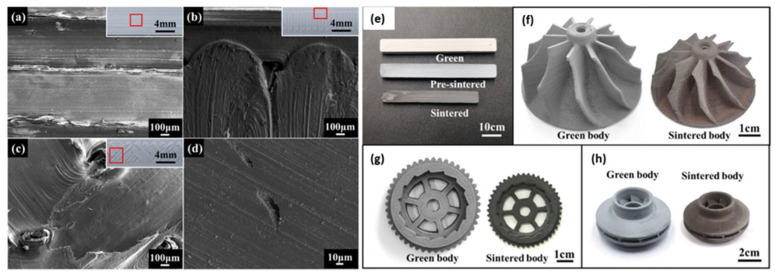
SEM images of printed layer: (**a**) contour offset path; (**b**) parallel lines path; (**c**) grid path; (**d**) ground surface of the sintered part by grid printing path. Images of Si_3_N_4_ parts prepared by MEX: (**e**) rectangular bar; (**f**) turbine rotor; (**g**) gear; (**h**) swirl fan [27].

Furong et al. [28] studied the fabrication of dense Si_3_N_4_ ceramics using the filament-based MEX method and gas pressure sintering. Continuous and stable extrusion, fundamental for MEX, relied on factors like the rheology performance of the feedstock, extrusion mechanism, and extrusion parameters. The feedstock exhibited shear-thinning behavior, which aided in smooth extrusion. The optimization of the nozzle diameter and layer thickness for smooth and uniform extrusion was carried out. Different layer thicknesses were explored, and challenges related to maintaining layer stability along the *z*-axis were acknowledged. Support structures in 3D printing were found to be necessary, affecting material consumption and surface quality. Experiments with printing bridge structures without support were completed, achieving varying success based on nozzle diameter and other factors. Material properties, including apparent density and flexure strength of Si_3_N_4_ ceramics, were evaluated. Apparent density remained relatively consistent across various printing parameters (99% on sintered parts). At the same time, flexure strength was influenced by the printing path, with contour offset and parallel lines paths showing higher strength than the grid path. The highest flexural strength achieved was 824.74 ± 85 MPa, associated with specific conditions such as layer thickness of 0.15 mm, nozzle size of 0.6 mm, and the contour offset path. Flexural strengths of 774.48 ± 75 MPa and 733.05 ± 70 MPa were attained when employing the contour offset and parallel lines paths, respectively. However, the grid path yielded a lower flexural strength of only 458.88 ± 40 MPa. The analysis of the Weibull modulus used for material reliability revealed that a high flexural strength did not necessarily correlate with a high Weibull modulus. Materials produced using the grid printing path exhibit a higher Weibull modulus at lower strength levels. Notably, even though materials with strength exceeding 700 MPa could be achieved through contour offset and grid printing paths, the Weibull modulus for these two paths was lower, specifically at 7.4 and 6.5, respectively. Inter-road bonding in inner-layer structures was explored through different printing paths. SEM images revealed variations in inter-road orientation and the presence of voids, potentially impacting material strength (Figure 12).

**Figure 13:**
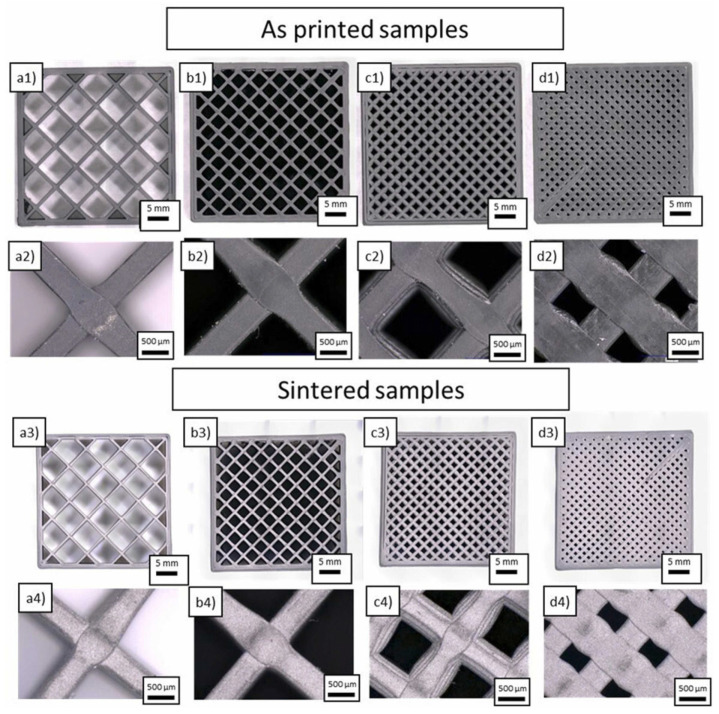
Sample realized from boron carbide (B_4_C) filament. Different infill value: (**a1**,**a2**) 20%, (**b1**,**b2**) 40%, (**c1**,**c2**) 60%, (**d1**,**d2**) 80%. Same sample after debinding and sintering at 2300 °C are shown with infill values of (**a3**,**a4**) 20%, (**b3**,**b4**) 40%, (**c3**,**c4**) 60%, (**d3**,**d4**) 80% [27].

Vozárová et al. [27] developed a composite filament of 65% micron-sized boron carbide powder dispersed in a thermoplastic binder, using a commercial FFF desktop printer with a 0.4 mm nozzle to create intricate green body structures. This process resulted in nearly fully dense boron carbide ceramics with part sizes of up to 4 cm and a relative density exceeding 96% after sintering (Figure 13). DTA/TG analysis was employed to optimize the process and determine the critical debinding temperature, set at 140 °C due to the thermal decomposition of the binder. Microstructural analysis using SEM images exhibited exceptional material homogeneity, while micro-CT images showcased precise replication of the experimental shapes, such as collimator-like printed grids. X-ray diffraction confirmed the presence of boron carbide with a minimal amount of free carbon phase (around 1% wt), which did not significantly affect the hardness value (29.88 ± 1.27 GPa). The binder system employed consisted of the Kuraray POVAL thermoplastic polymer with additives. Eliminating the binder resulted in superior material characteristics with no cracks or bubbles. Compared to conventionally cold-pressed boron carbide samples sintered under the same conditions, the samples had relative densities of around 96% and a well-defined microstructure after sintering. This study demonstrates the successful use of AM technologies to shape tough materials such as boron carbide.

## 3. Ceramic Materials as Reinforcement for Composite Parts

Wang et al. [52] focused their work on AM of ceramics, investigating the creation of fiber-reinforced ceramic matrix composites (FRCMCs). Unlike other extrusion techniques, filament-based MEX used heat to melt wire-based materials, while others relied on different mechanisms for solidification, such as chemical reactions. FRCMCs blend ceramic properties and fiber toughness, making them valuable in engineering. However, their performance needed to be improved for practical applications when fabricated through AM. Therefore, three main areas for improvement were identified:

- *Enhancing Mechanical Properties*: The minimization of defects and improved bonding between fibers and matrices were critical topics. Understanding how fiber characteristics and content influence composite properties was crucial for successfully applying these materials.
- *Developing Novel AM Technologies*: The current focus was primarily on short-fiber-reinforced ceramic matrix composites, but there was a shift toward continuous fiber reinforcement due to its superior properties.
- *Expanding Applications*: To leverage FRCMCs in various applications, developing advanced materials suitable for AM was crucial. These materials will be integrated into structural design and simulation analyses to create customized functionalities, ultimately achieving the seamless integration of structure and function in FRCMCs.

The research conducted by Freudenberg et al. [53] aimed to harness the mechanical and chemical-physical properties of techno-polymers [54,55] to develop ceramic matrix composites (CMCs) for aerospace applications. This exploration answered the need for advanced materials capable of meeting the rigorous demands of the aerospace industry, characterized by extreme environmental conditions and requirements for lightweight, strength, and durability. A novel technique for AM employed filament-based MEX with C-fiber (C/C-SiC) reinforced thermoplastic PEEK filaments (CF-PEEK). Thermal treatment at 325 °C for 48 h was applied to crosslink CF-PEEK to maintain sample shape during pyrolysis and prevent PEEK melting. Degassing channels and an open-porosity microstructure were printed for volatile product removal during PEEK pyrolysis and silicon infiltration. Near-net-shaped bending samples and large parts like gearwheels (up to 115 mm diameter) were fabricated using FFF with final crosslinking. Carbon short fibers (20 wt.%) protected by the carbon matrix hindered shrinkage, resulting in C/C-SiC samples with low open porosities (<1%) and flexural strengths up to 59 MPa. Fiber orientation affected strength, with the 90° alignment reducing strength by approximately 85%. These results demonstrated the potential for implementing this technique in continuous fiber-reinforced ceramic matrix composites (CMC).

Petousis et al. [56] explored the potential of titanium nitride (TiN) ceramic as a reinforcement agent for polylactic acid (PLA) thermoplastics. TiN, known for its extreme hardness, was commonly used in thin, hard coatings. The aim was to investigate the improvement in the mechanical properties of PLA due to the presence of nano-powder TiN. The resulting nanocomposite filaments were used to print specimens for mechanical testing and to study their thermal, structural, and morphological properties. The addition of TiN nanoparticles significantly enhanced the mechanical performance of PLA. Specifically, a 43.4% improvement in tensile strength and a 51.5% increase in flexural strength were observed for specimens containing 4 wt.% TiN loading. The study also evaluated the cost-effectiveness of the process, concluding that adding TiN did not introduce processability issues and was economically viable.

The same authors [57] extended research to explore the feasibility of developing composites using acrylonitrile butadiene styrene (ABS) combined with Si_3_N_4_ nanoparticles. Results demonstrated a notable enhancement in the mechanical properties of the composite material due to the addition of Si_3_N_4_ nanoparticles at a weight concentration of 4.0 wt.%. The tensile toughness showed the most substantial improvement, with a 56% increase compared to pure ABS. The tensile strength and tensile modulus of elasticity also improved by 25.6% and 20.2%, respectively. The flexural properties displayed enhanced values, with a 30.3% increase in flexural strength, a 47.2% increase in flexural toughness, and a 17.3% increase in flexural modulus of elasticity. Compression properties also showed enhancements, with a 29.4% increase in compression strength, a 34.3% increase in compression toughness, and a 21.5% increase in compression modulus of elasticity. The microhardness of the nanocomposites displayed a gradual improvement with increasing Si_3_N_4_ content, reaching a maximum enhancement of 34.9%, with the hardness of the ceramic nano additives playing a significant role in this enhancement. Thermogravimetric analysis indicated that the thermal stability of the thermoplastic polymer (ABS) was not significantly affected by the inclusion of Si_3_N_4_ nanoparticles. This finding supported the compatibility of the materials used in the study with the employed methodology and process conditions. The highest temperature utilized in the 3D printing process was considerably lower than the temperature at which significant weight loss begins (390 °C). Raman spectroscopy analyses did not detect any changes in the chemical bonds of the polymeric matrix, nor did they reveal any chemical reactions between ABS and Si_3_N_4_ nanoparticles. The results suggest the potential for scaling up the production of nanocomposites with an ABS matrix featuring enhanced mechanical performance through a cost-effective and straightforward approach suitable for industrial applications.

Vidakis et al. [58] explored the potential of using tungsten carbide (WC), a super-hard and thermally stable ceramic, as a reinforcement and stabilizing agent in ABS for MEX 3D printing. ABS/WC nanocomposite filaments were prepared with varying levels of WC content. Different tests were conducted to assess the impact of WC nano powder content on various characteristics of the nanocomposite filaments and 3D-printed samples. The experiments revealed that the nanocomposites exhibit excellent thermal stability and non-Newtonian shear thinning behavior, making them promising for processing [59]. WC nanoparticles significantly enhance the mechanical properties of the optimized nanocomposites compared to pure ABS. Specifically, ABS with 4.0 wt.% WC shows substantial increases in compressive, flexural, and tensile strength by 25.9%, 29.4%, and 20.9%, respectively, along with a remarkable 100.3% increase in microhardness, indicating a potential for high wear resistance applications. It successfully demonstrates that adding WC ceramic powder improves the mechanical characteristics of ABS. While the research primarily focuses on the reinforcement effect of WC nanoparticles on ABS, it also suggests future directions for investigation, such as studying the impact of WC nanoparticles on the aging of ABS polymers.

## 4. Conclusions

The filament-based 3D printing of ceramics was investigated, revealing significant developments and advancements in ceramic material extrusion technology. New horizons in the fabrication of complex ceramic components were opened. A key trend observed was the ongoing optimization of printing parameters, including nozzle size, layer thickness, and printing path, to enhance the quality and performance of ceramic components. These parameters significantly impacted the balance between ceramic materials’ strength and apparent density. The challenge of support structures during the printing process also persisted, affecting material consumption and surface quality. Various strategies to minimize support usage, including attempts to print unsupported bridge structures, were explored. Mechanical properties such as flexural strength underwent close evaluation, revealing significant influence from the printing path. However, flexural strength was not the sole indicator of the reliability of 3D-printed materials; the introduction of the Weibull modulus provided a measure of their reliability.

Finally, open challenges in the 3D printing of ceramics, including the need to address printing defects such as voids and suboptimal layer orientations, were highlighted. Future research should focus on advanced strategies to control and eliminate such defects, further enhancing the quality and reliability of ceramic components. In summary, filament-based 3D printing of ceramics witnessed significant progress and opened new perspectives in the fabrication of complex ceramic components. Considerable challenges remained, offering ongoing opportunities for future research and development in this promising field.

The emergence of AM pointed out the immense potential of the ceramic industry with the extensive use of AM, extending fields to new applications. However, AM should not be compared with conventional technologies based on cost, lead time, and performance comparison. Ceramics AM will push the current limits of conventional manufacturing, opening new markets with improved product density and performance.

## Figures and Tables

**Table 1:** Overview of ceramics obtained by filament-based MEX technology: feedstocks composition, printing parameters, and shrinkage data.

Material	Feedstock	Powder Particle Size d_50_ [μm]	Printing Parameters					Shrinkage (avg.)		References
			d [mm]	s [mm/s]	l_h_ [mm]	T_p_ [°C]	T_b_ [°C]			
Al_2_O_3_	▪ 99.8% pure Al_2_O_3_ powder (CT3000 Almatis GmbH, Ludwigshafen am Rhein, Germany) ▪ EVA 420 (DuPont, Wilmington, DE, USA) ▪ Stearic acid (Sigma Aldrich, Buchs, Switzerland)	0.5	0.6	10	-	30–170	-	height 23% diameter 18% wall thickness 12%		Gorjan et al. [19]
	▪ alumina filament (Fabru GmbH, Hinwil, Switzerland). ▪ Ethylene-vinyl acetate copolymer (EVA)	-	0.4	20	0.20	180	50	22.40%		Hadian et al., 2023 [20]
	▪ Al_2_O_3_ (TM-DAR, Taimai Chemicals, Tokyo, Japan) ▪ Polyvinyl butyral (Mowital B30H, Kuraray Europe GmbH, Hattersheim am Main, Germany) ▪ Polyethyleneglycol (PEG 4000, Roth GmbH, Karlsruhe, Germany) ▪ Stearic acid (SA, Roth GmbH, Karlsruhe, Germany)	0.1	0.25	10	0.10	165	60	20.75%		Nötzel et al., 2020 [21]
	▪ alumina powder (99.99%) (Sumitomo Chemical Co. Ltd., Tokyo, Japan) [solid load 80 wt.%] ▪ polyolefin-based binder system	0.4–0.7	0.4	10–15	0.10	240	60	20% 19%		Orlovská et al., 2020 [22] Orlovská et al., 2021 [23]
	▪ alumina powder (Plasmotherm Ltd., Moscow, Russia) ▪ PLA powder (eSun Ltd., Shenzhen, China)	30	0.8	-	0.40	220	100			Smirnov et al. [24]
	▪ Alumina Zetamix (Nanoe, Ballainvilliers, France) [solid loading 83%wt] ▪ Binder system	<1.0	0.6	20	0.2	150	50	20.70%		Tosto et al. [25]
			-	30	0.20	150	25	22.50%		Truxová et al. [26]
B_4_C	▪ high-purity boron carbide powder [solid loading 65%wt] ▪ Kuraray POVAL thermoplastic polymer (Kuraray Europe GmbH)	3	0.4	10	-	210	50	19.41%		Vozárová et al. [27]
Si_3_N_4_	▪ Si_3_N_4_ powder (Jinsheng Ceramic Technology Co., Ltd., Changzhou, China) ▪ Al_2_O_3_ and Y_2_O_3_ as sintering additives ▪ Ethylene-vinyl acetate copolymer (Beijing Organic Chemical Company, Beijing, China) ▪ Polyethylene (Polymeric Chemicals Co. Ltd., Zhejiang, China) ▪ Paraffin wax (Jingmen Petrochemical Company, Jingmen, China) ▪ Stearic acid (Delun Chemical Technology Co. Ltd., Xiamen, China)	0.69	0.4, 0.6, 0.8	25	0.10, 0.15, 0.20	170	80	24.20%		Furong et al. [28]
ZrO2	▪ 3Y-TZP filament (Zetamix White Zirconia, Nanoe, France)	0.3	0.4	-	-	-	-	-		Bhandari et al. [29]
			0.6	-	0.15	200	-	18%		Petit et al. [30]
	▪ TZ-3YS-E (Tosoh Europe B.V., Amsterdam, The Netherlands) ▪ TPE, Kraiburg TPE GmbH & Co. KG, Waldkraiburg, Germany) ▪ Polyolefin grafted with a polar component (gPO, BYK Chemie GmbH, Wesel, Germany).	0.09	0.6	12.5	0.15	255	100	20%		Cano et al. [31]
	▪ YSZ filament SiCeram GmbH (SiCeram GmbH, Jena, Germany) ▪ YSZ filament PT+ A GmbH (PT+ A GmbH) ▪ YSZ filament Fabru GmbH (Fabru GmbH)	-	0.6	30	0.2	145 180	45 40	Only Fabru filament sintered 24%		Clemens et al. [32]
	▪ OZ-3Y powder (Guangdong Orient ZirconiaInd. Sci & Tech Co., Ltd., Yanhong Town, China) ▪ High-density PE (Polymeric Chemicals Co. Ltd., China) ▪ SEBS (Kraton Polymers Inc., The Woodlands, TX, USA) ▪ Paraffin wax (Jingmen Petrochemical Company, Jingmen, China) ▪ Stearic acid (Delun Chemical Technology Co. Ltd., China) ▪ Dibutyl phthalate (Sinopharm Chemical Reagents Co., Ltd., Shanghai, China)	0.5	0.6	12	-	-	-	wt.% 78% 80% 82%	shrink. 29.7% 28.4% 26.7%	Guan et al. [33]
ZrO_2_	▪ TZ-3YS-E (Tosoh, Japan) ▪ Elvax 460 (18% VA, DuPont, USA) ▪ Elvax 420 (18% VA, 18% VA, DuPont, USA) ▪ Stearic acid (Sigma Aldrich)	0.6	0.8	8	-	180	60	23%		Hadian et al., 2021 [34]
	▪ TZ-3YS-E (Tosoh, Tokyo, Japan) ▪ PIM binders, Embemould K83G, Embemould K84G, Embemould M, and Embemould CC (KRAHN Chemie, Hamburg, Germany) ▪ Commercial YSZ filament (Fabru GmbH, Switzerland)	0.6	0.8	25	0.40	180	40	21.5%		Hadian et al., 2022 [35]
	▪ 3Y-TZP OZ-3Y powder (Guangdong Orient Zirconic Ind. Sci & Tech Co., Ltd., Shantou, China) [solid loading 85 wt.%] ▪ Ethylene-vinyl acetate copolymer ▪ Polyethylene ▪ Paraffin wax ▪ Stearic acid	0.5	0.2–1.0	50–80	0.10	130–190	-	20%		He et al. [36]
	▪ TZ-3YS-E (Tosoh, Japan) [solid content 50 vol%] ▪ Paraffin wax (Sasolwax 6403, Sasol Wax GmbH, Hamburg, Germany) ▪ Low Density PE (Lupolen PE1800H, LyondellBasell, Bayreuth, Germany) ▪ Stearic acid (Roth GmbH, Karlsruhe, Germany)	1.04	0.4	10	0.10	170	70	20.90%		Nötzel et al., 2021 [37]
ZrO_2_—17-4PH Multi-material	▪ Tetragonal yttria-stabilized zirconia ▪ TPE binder ▪ Polyolefin ▪ Stearic acid	0.50	-	10	-	220	20	-		Abel et al. [9]

**Table 2:** Comparison table for the mechanical properties of ceramics obtained by filament-based MEX technology.

Material	Mechanical Test	Flexural Strength σ [MPa]		Characteristic Flexural Strength σ_0_ [MPa]		Weibull Modulus m	References
Al_2_O_3_	Biaxial flexural test				145.5	3.0	Gorjan et al. [19]
	3-point bending test	l_h_ 0.3 mm l_h_ 0.2 mm l_h_ 0.1 mm	200 260 300	-		-	Orlovská et al., 2020 [22]
	3-point bending test	232.6		-		-	Tosto et al. [25]
	3-point bending test	Infill 80% Infill 90% Infill 100%	316.12 327.24 331.61	-		-	Truxová et al. [26]
Si_3_N_4_	Biaxial flexural test	-			824.74	7.0	Furong et al. [28]
ZrO_2_	3-point bending test	raster 0° raster +/− 45° raster 90°	512 473 366		537 508 404	9.9 6.5 4.3	Cano et al. [31]
	3-point bending test	wt.% 78% wt.% 80% wt.% 82%	206.3 311.1 492.8	-		-	Guan et al. [33]
	Biaxial flexural test	-			91	5.7	Hadian et al., 2021 [34]
	Biaxial flexural test	-		feedstock filament	203 531	4.3 3.5	Hadian et al., 2022 [35]
	3-point bending test		890	-		-	He et al. [36]
ZrO_2_ 5Y-PSZ (Ceramill ZOLID FX, Herrschaftswiesen, Austria) traditionally manufactured as a reference	Biaxial flexural test	-		as fired polished	633.8 943.9	9.8 6.6	Spintzyk et al. [38]

## Data Availability

Not applicable.
